# Vonoprazan and *Helicobacter pylori* Treatment: A Lesson From Japan or a Limited Geographic Phenomenon?

**DOI:** 10.3389/fphar.2019.00316

**Published:** 2019-04-05

**Authors:** Amin Talebi Bezmin Abadi, Enzo Ierardi

**Affiliations:** ^1^Department of Bacteriology, Faculty of Medical Sciences, Tarbiat Modares University, Tehran, Iran; ^2^Section of Gastroenterology, Department of Emergency and Organ Transplantation, University of Bari Aldo Moro, Bari, Italy

**Keywords:** *Helicobacter pylori*, therapy, PPI, treatment, vonoprazan

## Abstract

Within a short time after the discovery of *Helicobacter pylori*, its critical role in many gastroduodenal disorders became evident. Many *in vitro* and *in vivo* data have proven that infection should be treated in order to avoid lasting colonization which may lead to problematic gastroduodenal diseases. Probiotics, preventive and therapeutic vaccines and antibiotic therapy are the main options proposed to cure these disorders. 25 years ago, triple therapy including a traditional proton pump inhibitor (PPI) and two antibiotics (amoxicillin and clarithromycin or metronidazole) was defined as the best therapy formulation for the *H. pylori* infection. With the strongly decreased effectiveness of this scheme, many empirical therapeutic regimens have been developed. However, the prevalence of resistance is increasing worldwide and reveals important geographic differences and even the most recent and effective regimens show some critical points. Therefore, efficacy of vonoprazan-based therapy in regions with low rate of clarithromycin resistance may be limited. In this review, we attempt to open a new window to overcome the problem of antibiotic resistance to *H. pylori*. In fact, we focused our attention on the possibility that conventional PPI may be replaced by vonoprazan, thus giving rise to the beginning of a new era characterized by an improved therapeutic option for *H. pylori* infection. Therefore, we hypothesize that switching to vonoprazan as a novel acid blocker for *H. pylori* treatment might allow an unexpected reassessment of the triple therapy, at least in regions with low rate of clarithromycin resistance. Nevertheless, this optimistic view of the problem could be disproved by the possible failure of vonoprazan based therapeutic regimens outside of Japan in geographic areas characterized by different rates of antibiotic resistances.

## Introduction

*Helicobacter pylori* (*H. pylori*) is a bacterium that induces chronic gastric inflammation and is the most important cause of peptic ulcer worldwide. Bacterial colonization mostly remains active lifelong if an effective therapy is not applied. *H. pylori* has been demonstrated to be susceptible to some common antibiotics *in vitro*, therefore, a successful eradication should not be difficult. However, recently, *H. pylori* infection has become difficult to treat. The failure of the therapy is mainly due to the presence of antibiotic-resistant organisms. Despite the fact that there is a general agreement that the main reason for eradication failure is the rapid increase in bacterial antibiotic resistance, poor patient compliance and rapid metabolism of conventional PPIs might also be crucial elements of this relevant problem, which could have not been properly considered in this setting.

## What Has Been Done to Solve This Problem?

International guidelines (Table 1) discouraged clarithromycin and levofloxacin use in areas with resistance rates higher than 15% (Chey, 2012; Malfertheiner et al., 2012, 2016; Kim et al., 2013; Lee, 2014; Sugano et al., 2015; Zagari et al., 2015; Fallone et al., 2016). However, it is known that guidelines are concentrated on the clinical practice in the context of empirical settings. Consequently, first line schedules with expected high eradication levels, i.e., bismuth containing quadruple therapy or non-bismuth concomitant quadruple therapy, are suggested (Zagari et al., 2015). However, the 2009 Asia-Pacific guidelines highlighted a similar efficacy for both 14-day triple therapy and bismuth containing quadruple in first line (Fock et al., 2009). We here emphasize that quadruple therapies require daily assumption of an excessive number of tablets (14 and 8 for bismuth and non-bismuth containing schedules, respectively) (Lee et al., 1999). Therefore, an evident doubt should be raised about the compliance. A complete adherence may be expected by subjects who are persuaded by major conditions, such as MALT lymphoma, family history of gastric cancer or peptic ulcer, particularly when complicated by episodes of bleeding, and the need for a long-term assumption of drugs able to induce gastric lesions, such as non-steroidal anti-inflammatory ones. Conversely, the majority of *H. pylori* positive subjects are dyspeptic or asymptomatic and could not be driven by convincing motivations, since the eradication of the bacterium is often not accompanied by a clinical benefit (Zullo et al., 2014). An incomplete adherence to an antibiotic therapy may be an important reason of resistance onset, since it stimulates resistant mutant development (Megraud et al., 2013). Moreover, bismuth containing quadruple therapy includes the use of tetracycline, which shows a very low resistance in Europe (1–5%) (Ierardi et al., 2013). Nonetheless, in Asia, even tetracycline resistance rates of 19% have been reported (Graham and Lee, 2015). So, in the near future an increase of resistance to this antibiotic could be consequent to its use on a large scale with an incomplete patient adherence (Graham et al., 2014). Additionally, metronidazole resistance is generally estimated to be no lower than 50% in Asia. Concomitant therapy implies the use of three conventional antibiotics with the evident aim to overcome the resistance to each single drug due to the overall combined effect of the regimen. Presumably, for this reason, concomitant schedule is suggested as the treatment of choice, when compared to sequential therapy (Losurdo et al., 2016). A final point concerns the duration of suggested first line therapies. The commercial kit of bismuth containing quadruple therapy provides a number of pills necessary for a 10-day schedule and the same period is the minimal time requested for concomitant regimen. Nevertheless, a prolongation to 14 days of both treatments seems to improve their effectiveness (Fallone et al., 2016; Malfertheiner et al., 2016). Interestingly, in a recent systematic review and meta-analysis Gao et al. (2016) showed that high-dose PPI-amoxicillin dual therapy is comparable to recommended rescue therapies for *H. pylori* infection. On these bases, the possibility of improving *H. pylori* treatment outcome with new PPIs able to increase the effectiveness of antibiotics in gastric microniche should open a new scenario. This action could be a rescue solution in order to help clinicians to restart the war against gastric cancer and other *H. pylori*-related diseases.

**Table 1:** Published guidelines about management of *Helicobacter pylori* using conventional proton pump inhibitors (PPI).

Authors	Journal	Year	Title	References
Fallone et al.	Gastroenterology	2016	The Toronto Consensus for the Treatment of Helicobacter pylori Infection in Adults	Fallone et al., 2016
Sheu et al.	Helicobacter	2017	Consensus on the clinical management, screening-to-treat, and surveillance of H. pylori infection to improve gastric cancer control on a nationwide scale	Sheu et al., 2017
Zagari et al.	Digestive and Liver Disease	2015	Guidelines for the management of Helicobacter pylori infection in Italy: The III Working Group Consensus Report 2015	Zagari et al., 2015
Malfertheiner et al.	GUT	2012	Management of Helicobacter pylori infection: the Maastricht IV/ Florence Consensus Report	Malfertheiner et al., 2012
Malfertheiner et al.	GUT	2007	Current concepts in the management of Helicobacter pylori infection: the Maastricht III Consensus Report	Malfertheiner et al., 2007
Malfertheiner et al.	Aliment Pharmacol Ther	2002	Current concepts in the management of Helicobacter pylori infection: The Maastricht 2-2000 Consensus Report	Malfertheiner et al., 2002
Malfertheiner et al.	GUT	1997	Current European concepts in the management of Helicobacter pylori infection. The Maastricht Consensus Report	Malfertheiner et al., 1997
Malfertheiner et al.	GUT	2017	European Helicobacter and Microbiota Study Group and Consensus panel. Management of Helicobacter pylori infection-the Maastricht V/Florence Consensus Report	Malfertheiner et al., 2017


## Proton Pump Inhibitors in Eradication Regimens of *H. pylori*

Proton pump inhibitors (PPIs) are commonly used in all therapeutic lines for eradication of *H. pylori*. There is no empiric anti-*H. pylori* therapeutic regimen able to eradicate 100% of infections and, therefore, researchers constantly attempt to find better formulations. At the time of the first eradication attempts, PPI-based therapy was found to show a higher efficacy in comparison with non-PPI-based therapy. Therefore, the prescription of anti-secretory drugs is considered necessary for the efficacy of antibiotic therapy. The entire impact of this procedure is due to the following reasons. First, induced hypochloridria is a phenomenon which indirectly influences successful colonization of *H. pylori* in gastric territory, thus the bactericidal effects of available antibiotics can be easily observed (McColl, 2012). Additionally, current evidence insists on the role of hypochloridria for enforcing the deadly effect on the bacterium colonization in the stomach. Moreover, the addition of lansoprazole (as major conventional antisecretory drug) in culture media reduces the survival rate of the *H. pylori*. Hypothetically, this event can happen also in the gastric mucosa (Hagiwara et al., 2015). Therefore, it can be asserted that increasing the concentration of lansoprazole drastically reduces the *H. pylori* survival at normal acidity. Moreover, there are some reports showing that, at least for amoxicillin, the minimal inhibitory concentration (MIC) is increased in relation to a high pH following the presence of antisecretory drug. Analogously, increasing pH can highly improve the bactericidal effects of clarithromycin in gastric juice. On the other hand, long term use of conventional PPIs may induce or worsen atrophic gastritis in patients colonized with *H. pylori* (Hagiwara et al., 2015; Sheu et al., 2017). Therefore, long-term use of PPIs in *H. pylori* positive patients is discouraged. In this context, we believe that the availability of vonoprazan as an alternative to PPIs is relevant in empiric therapy against *H. pylori* infection.

## Vonoprazan Advantages Compared to Other PPIs

**FIGURE 1:**
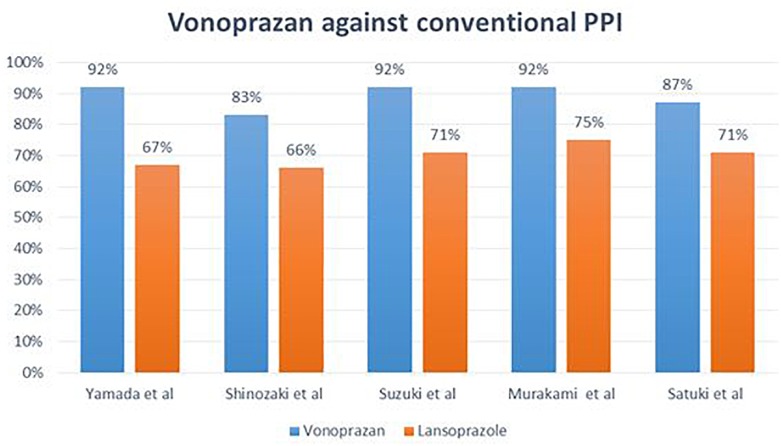
Different eradication rates observed following application of vonoprazan versus conventional PPIs.

There are some speculative arguments in favor of the application of vonoprazan instead of other PPIs as antisecretory drugs: (1) it is more potent in the inhibition of acid secretion, (2) it has a fast onset of action, (3) less antisecretory variation is reported, as well as (4) greater safety, and (5) better tolerability. In brief, pharmacodynamic characteristics of vonoprazan clearly suggest a better performance when compared to conventional PPIs. In Figure 1, eradication rates following the application of vonoprazan versus conventional PPIs in triple therapy regimens against *H. pylori* are reported. In three retrospective studies carried out between 2013 and 2015 (Yamada et al., 2016; Suzuki and Hideki, 2018), the efficacies of vonoprazan 20 mg bid, amoxicillin 750 mg bid, clarithromycin 200 mg bid for a period of 7 days were examined. ^13^C-urea breath test, as recommended by current guidelines, was the confirmation test in those studies. In the only available randomized clinical trial by Murakami et al. (2016) vonoprazan 20 mg bid, amoxicillin 750 mg bid and clarithromycin 200 or 400 mg bid were used for 7 days. Similar to other studies, UBT was applied to confirm the successful eradication of *H. pylori* infection. The results of these studies are summarized in Figure 1. The results of these studies and the related meta-analysis report a very interesting and surprising finding: the possibility that an old therapeutic regimen, which all current guidelines suggest should be removed from the clinical practice because it is outdated and ineffective, can be effective again when combined with a new modality of acid-inhibition.

## Discussion

Until 2014, most of the trials were devoted to designing better therapies for *H. Pylori* infection by using various antibiotics in formulation of triple or quadruple therapies; however, some reports focused the attention on a possible relevant role of PPIs in eradicating regimens (Sugimoto and Yamaoka, 2018). With this regard, a pioneering strategy has been initiated in Japan by the advent of a new PPI, i.e., vonoprazan (Sugimoto and Yamaoka, 2018). The sharp decrease in eradication rates of therapies using conventional PPI was the main rationale for introducing vonoprazan in the clinical practice (Ashida et al., 2016). As a whole, the actual value of conventional PPIs being debated for the following reasons: (i) slow onset of pharmacological action in acidic gastric microniche, (ii) instability in acidic condition of human stomach and (iii) problematic variations observed in genetic polymorphisms of cytochrome P450 (CYP) 2C19 metabolism. Additionally, recent evidence demonstrates that high-dose PPI-amoxicillin dual therapy is comparable to recommended rescue therapies (Tai et al., 2019). Presumably, the set of all these problems stimulated the researchers to develop the new generation of PPI called “Vonoprazan fumarate” (Takecab^TM^). Since 2015, it has become a first-in-class potassium-competitive acid blocker in Japan (Garnock-Jones, 2015). Vonoprazan shows a bioactivity 300 times greater when compared with lansoprazole (Garnock-Jones, 2015; Ashida et al., 2016; Graham, 2016; Maruyama et al., 2017). Similar to PPIs, vonoprazan reduces gastric hydrogen/potassium acid-inhibitory effects even if it shows a higher stability in gastric harsh conditions. This property may explain its effects in improving the efficacy rate of conventional triple therapy for *H. pylori* infection. *H. pylori* therapy was covered by the Japanese insurance system, thus more patients were admitted to receive therapy including vonoprazan in this country with the possibility of performing clinical studies regarding its efficacy. Recently, Sakurai et al. (2017) showed the effectiveness of vonoprazan-based *H. pylori* eradication therapy among Japanese populations; they found Vonoprazan-based therapy to be more effective than conventional PPI-based therapy, at least in clinical settings. Moreover, Sue et al. (2017) reported that the eradication rate of first-line therapy using vonoprazan-based amoxicillin and clarithromycin in a week is superior to conventional PPI-based therapy. The problem with this study was that the eradication rate was around 90%. The promising point in their study was the well-tolerated rate of vonoprazan-based therapy in patients. Recently, Saito et al. (2019) have shown that the dual therapy using vonoprazan and amoxicillin can brings a higher eradication rate than standard *H. pylori* treatment. With this regard, in a Randomized Control Trial, Ozaki et al. (2018) concluded that vonoprazan is the future of successful therapy of *H. pylori* in different regions. Again, this research group confirmed that there is no adverse side effect due to the application of vonoprazan in therapeutic regimens of *H. pylori*. In order to examine the effect of vonoprazan in third-line therapy, Sue et al. (2018) have shown that a 7-day triple therapy containing vonoprazan, amoxicillin, and sitafloxacin brings higher efficacy rate when compared with a combined regimen with PPI, sitafloxacin and amoxicillin. To the date of writing this paper (February 2019), there are no other published trials examining the efficacy of vonoprazan in *H. pylori* infected populations in countries other than Japan. So far, many PPIs have been investigated in clinical trials, but the newly introduced acid inhibitor vonoprazan brought a glimpse of hope to have a higher efficacy in treating the *H. pylori* infection. In a meta-analysis by Jung et al. (2017) authors showed many benefits of the vonoprazan-based triple therapy in comparison with the same regimen including conventional PPIs. However, this analysis included retrospective observational studies thus raising a serious problem about the real suitability of this meta-analysis. Nevertheless, the finding could support speculative considerations, i. e. switching to vonoprazan as novel acid blocker can initiate a new era for of *H. pylori* treatment with an unexpected reassessment of triple therapy, at least in regions with low rate of clarithromycin resistance (Maruyama et al., 2017). Therefore, a prospective study using vonoprazan in other regions is needed in order to generalize the suggestion of promoting vonoprazan as the most useful anti-acid in the combination of antibiotics against *H. pylori* thus avoiding that a limited geographic phenomenon can become a confusing paradigm to be applied worldwide.

## Future Perspective

After two decades, it seems that the era of conventional PPI may be ending and the one of vonoprazan is beginning in order to obtain a better therapy for *H. pylori* infection. Nevertheless, the newly proposed regimen including vonoprazan should be evaluated in countries different from Japan in order to consider it as a global drug. In conclusion, based on the increased antibiotic resistance rates, the scarceness of hope for the arrival of new antibiotics and the poor enthusiasm of companies funding the development of an efficient vaccine, vonoprazan-based regimens may have disclosed an excellent option to test. The lack of knowledge concerning the effectiveness of vonoprazan in other therapeutic regimens is a gap which could be filled by future studies (Jung et al., 2017). Furthermore, the actual effectiveness of vonoprazan in regions with high rate of clarithromycin resistance (more than 20%) is not clear yet; this is another crucial point. Nevertheless, according to current knowledge, strategies which aim to maintain a high gastric pH may overcome the problem of antibiotic resistance hampering a successful *H. pylori* therapy. This solution may be translated in vonoprazan. Conclusively, switching to using vonoprazan as novel acid blocker may be a stimulating topic for the near future since it could disclose a new scenario in *H. pylori* treatment.

## Author Contributions

All authors listed have made a substantial, direct and intellectual contribution to the work, and approved it for publication.

## Conflict of Interest Statement

The authors declare that the research was conducted in the absence of any commercial or financial relationships that could be construed as a potential conflict of interest.
